# Trends in survival among extremely-low-birth-weight infants (less than 1000 g) without significant bronchopulmonary dysplasia

**DOI:** 10.1186/1471-2431-12-63

**Published:** 2012-06-08

**Authors:** Francesc Botet, Josep Figueras-Aloy, Xavier Miracle-Echegoyen, José Manuel Rodríguez-Miguélez, MªDolors Salvia-Roiges, Xavier Carbonell-Estrany

**Affiliations:** 1 Neonatology Service, Hospital Clínic de Barcelona, Sabino de Arana 1, 08028 Barcelona, Spain; 2 Institut d’Investigacions Biomèdiques August Pi i Sunyer (IDIBAPS), Barcelona, Spain; 3 Universitat de Barcelona, Barcelona, Spain; 4 Hospital Clínic de Barcelona, Sabino de Arana 1, 08028 Barcelona, Spain

**Keywords:** Bronchopulmonary dysplasia, Chronic lung disease, Extremely low birth weight infants, Preterm infants

## Abstract

**Objective:**

The aim of this study was to analyze the evolution from 1997 to 2009 of survival without significant (moderate and severe) bronchopulmonary dysplasia (SWsBPD) in extremely-low-birth-weight (ELBW) infants and to determine the influence of changes in resuscitation, nutrition and mechanical ventilation on the survival rate.

**Study design:**

In this study, 415 premature infants with birth weights below 1000 g (ELBW) were divided into three chronological subgroups: 1997 to 2000 (n = 65), 2001 to 2005 (n = 178) and 2006 to 2009 (n = 172).

Between 1997 and 2000, respiratory resuscitation in the delivery room was performed via a bag and mask (Ambu®, Ballerup, Sweden) with 40-50% oxygen. If this procedure was not effective, oral endotracheal intubation was always performed. Pulse oximetry was never used. Starting on January 1, 2001, a change in the delivery room respiratory policy was established for ELBW infants. Oxygenation and heart rate were monitored using a pulse oximeter (Nellcor®) attached to the newborn’s right hand. If resuscitation was required, ventilation was performed using a face mask, and intermittent positive pressure was controlled via a ventilator (Babylog2, Drägger). In 2001, a policy of aggressive nutrition was also initiated with the early provision of parenteral amino acids. We used standardized parenteral nutrition to feed ELBW infants during the first 12–24 hours of life. Lipids were given on the first day. The glucose concentration administered was increased by 1 mg/kg/minute each day until levels reached 8 mg/kg/minute. Enteral nutrition was started with trophic feeding of milk. In 2006, volume guarantee treatment was instituted and administered together with synchronized intermittent mandatory ventilation (SIMV + VG). The complications of prematurity were treated similarly throughout the study period. Patent ductus arteriosus was only treated when hemodynamically significant. Surgical closure of the patent ductus arteriosus was performed when two courses of indomethacin or ibuprofen were not sufficient to close it.

Mild BPD were defined by a supplemental oxygen requirement at 28 days of life and moderate BPD if breathing room air or a need for <30% oxygen at 36 weeks postmenstrual age or discharge from the NICU, whichever came first. Severe BPD was defined by a supplemental oxygen requirement at 28 days of life and a need for greater than or equal to 30% oxygen use and/or positive pressure support (IPPV or nCPAP) at 36 weeks postmenstrual age or discharge, whichever came first. Moderate and severe BPD have been considered together as “significant BPD”. The goal of pulse oximetry was to maintain a hemoglobin saturation of between 88% and 93%. Patients were considered to not need oxygen supplementation when it could be permanently withdrawn. The distribution of the variables was not normal based on a Kolmogorov-Smirnov test (p < 0.05 in all cases). Therefore, quantitative variables were expressed as the median and interquartile range (IQR; 25^th^-75^th^ percentile). Statistical analysis of the data was performed using nonparametric techniques (Kruskal-Wallis test and Mann–Whitney *U* test). A chi-square analysis was used to analyze qualitative variables. Potential confounding variables were those possibly related to BPD in survivors (p between 0.05 and 0.3 in univariate analysis). Logistic regression analysis was performed with variables related to BPD in survivors (p < 0.05) and potential confounding variables. The forward stepwise method adjusted for confounding factors was used to select the variables, and the enter method using selected variables was used to obtain the odds ratios.

**Results and conclusion:**

There was an increase in the rate of SWsBPD (1997 to 2000: 58.5%; 2001 to 2005: 74.2%; and 2006 to 2009: 75.0%; p = 0.032). In survivors, the occurrence of significant BPD decreased after 2001 (9.5% vs. 2.3%; p = 0.013). The factors associated with improved SWsBPD were delivery by caesarean section, a reduced endotracheal intubation rate and a reduced duration of mechanical ventilation.While the mortality of ELBW infants has not changed since 2001, the frequency of SWsBPD has significantly increased (75.0%) in association with increased caesarean sections and reductions in the endotracheal intubation rate, as well as the duration of mechanical ventilation.

## Introduction

Bronchopulmonary dysplasia (BPD) is the most common cause of chronic lung disease and is one of the most important sequelae of premature birth. BPD was originally described in children born slightly premature with severe respiratory distress syndrome (RDS) who had been exposed to aggressive mechanical ventilation and high concentrations of inspired oxygen. In recent years, this phenotype has been replaced by a new form of BPD that occurs in infants who are much less mature, often resulting in less iatrogenic injury. In this form of BPD, the immature lung fails to reach its full structural complexity, resulting in a global reduction in the surface available for gas exchange [1-5].

We hypothesized that survival without significant (moderate and severe) BPD (SWsBPD) would be higher among a group of ELBW infants born between 1997 and 2009 and would be related to the primary changes that occurred during this period of time, namely, a new delivery room respiratory resuscitation strategy, an aggressive nutrition regimen and a new mode of mechanical ventilation.

## Patients and methods

The study group included all ELBW infants (less than 1000 g) born in our hospital between January 1997 and December 2009 (n = 415). The neonates were divided into three chronological subgroups according to birth date: group 1, 1997 to 2000 (n = 65); group 2, 2001 to 2005 (n = 178); and group 3, 2006 to 2009 (n = 172). The study was approved by the Ethics Committee in Neonatology of the Hospital Clínic de Barcelona.

Between 1997 and 2000, respiratory resuscitation in the delivery room was performed via a bag and mask (Ambu®, Ballerup, Sweden) with 40-50% oxygen. If this procedure was not effective, oral endotracheal intubation was always performed before chest compressions were applied. Pulse oximetry was never used. Starting on January 1, 2001, a change in the delivery room respiratory policy was established for ELBW infants. Oxygenation and heart rate were monitored using a pulse oximeter (Nellcor®) attached to the newborn’s right hand. If resuscitation was required, ventilation was performed using a face mask, and intermittent positive pressure was controlled via a ventilator (Babylog2, Drägger), which applied a peak inspiratory pressure (PIP) ranging from 20 to 25 cmH_2_O, a positive end-expiratory pressure (PEEP) of 6 cmH_2_O, a rate of 50 breaths per minute, and an inspiratory time of 0.4 seconds (inspiratory/expiratory ratio = 1/2). Oxygen concentrations were adjusted (Babymix2, Drägger) to maintain the oxygen saturation between 85% and 90%. Respiratory support after stabilization, during transport to the Neonatal Intensive Care Unit (NICU), and upon admission to the NICU was provided with continuous positive airway pressure (CPAP).

In 2001, a policy of aggressive nutrition [6,7] was also initiated with the early provision of parenteral amino acids [8,9]. All preterm infants were fed according to our protocol [10]. We used standardized parenteral nutrition (1.5 g/kg/day of protein and 5 mg/kg/minute of glucose in 70 mL/kg/day of water) to feed ELBW infants during the first 12–24 hours of life. Fluids were set at 70 mL/kg/day on the first day of life and then increased to 10–20 mL/kg/day until they reached 160 mL/kg/day on the 7^th^ or 8^th^ days of life. Lipids were given at 1 g/kg/day on the first day. Amino acid and lipid intake were increased by 0.5 g/kg/day until they reached 3 g/kg/day and 2.5 g/kg/day, respectively. The glucose concentration administered was increased by 1 mg/kg/minute each day until levels reached 8 mg/kg/minute. Insulin was added when serum glucose levels were >150 mg/dL, with glycosuria or serum glucose levels >180 mg/dL. Enteral nutrition was started with trophic feeding of milk at 4, 8, 16 and 32 mL/kg/day on days 1, 2, 3 and 4 of life, respectively; thereafter, enteral milk was increased by 20 mL/kg/day every day until levels reached 160 mL/kg/day on the 10^th^ day of life. Preterm formula was given at a concentration of 16% milk beginning on the first day of life, and if expressed breast milk was given, 3% fortification was added when half of the total enteral dose target was reached.

In 2006, volume guarantee treatment was instituted and administered together with synchronized intermittent mandatory ventilation (SIMV + VG). To achieve good alveolar recruitment, a PEEP between 5 and 7 cmH_2_O was applied. The initial target tidal volume is set between 4 and 5 mL/kg. Larger tidal volumes of up to 6 mL/kg may be required in extremely premature infants.

The complications of prematurity were treated similarly throughout the study period. Patent ductus arteriosus was only treated when hemodynamic repercussion (need for oxygen or CPAP/VPPI, severe pulmonary hypertension). Surgical closure of the patent ductus arteriosus was performed when two courses of indomethacin or ibuprofen were not sufficient to close it.

Mild BPD were defined by a supplemental oxygen requirement at 28 days of life and moderate BPD if breathing room air or a need for <30% oxygen at 36 weeks postmenstrual age or discharge from the NICU, whichever came first. Severe BPD was defined by a supplemental oxygen requirement at 28 days of life and a need for greater than or equal to 30% oxygen use and/or positive pressure support (IPPV or nCPAP) at 36 weeks postmenstrual age or discharge, whichever came first [2]. Moderate and severe BPD have been considered together as “significant BPD”. The goal of pulse oximetry was to maintain a hemoglobin saturation of between 88% and 93%. Patients were considered to not need oxygen supplementation when it could be permanently withdrawn. In groups 1 and 2, the classification was established retrospectively.

The distribution of the variables was not normal based on a Kolmogorov-Smirnov test (p < 0.05 in all cases). Therefore, quantitative variables were expressed as the median and interquartile range (IQR; 25^th^-75^th^ percentile). Statistical analysis of the data was performed using nonparametric techniques (Kruskal-Wallis test and Mann–Whitney *U* test). A chi-square analysis was used to analyze qualitative variables. Potential confounding variables were those possibly related to BPD in survivors (p between 0.05 and 0.3 in univariate analysis). Logistic regression analysis was performed with variables related to BPD in survivors (p < 0.05) and potential confounding variables. The forward stepwise method adjusted for confounding factors was used to select the variables, and the enter method using selected variables was used to obtain the odds ratios.

## Results

The characteristics of the newborns in the three groups are shown in Table 1. The initial severity of the infants’ clinical conditions in the three groups was similar, without significant differences in the CRIB score or the incidence of respiratory distress syndrome (Table 2). The morbidities studied, including those related to infections, necrotizing enterocolitis, intraventricular hemorrhage, and periventricular leukomalacia, were also similar between groups. The differences between the SWsBPD group and the group comprising deceased or surviving infants with significant BPD are shown in Table 3. Logistic regression analysis adjusted for confounding factors (Table 4) confirmed that the factors significantly and independently associated with an increase in SWsBPD included birth by cesarean section from a single pregnancy without the need for deep resuscitation, higher birth weight, little or no need for mechanical ventilation, and the absence of grade III-IV intraventricular hemorrhage and severe arterial hypotension.

**Table 1:** Characteristics of the ELBW infants in this study

	**Group 1**	**Group 2**	**Group 3**	p-value*
	**(1997–2000)**	**(2001–2005)**	**(2006–2009)**	
	**(N = 65)**	**(N = 178)**	**(N = 172)**	
Gestational age (w)	27.0 (26–28)	27.0 (25.6-29.0)	26.9 (25.7-28.8)	0.988
Birth weight (g)	814 (680–935)	800 (700–915)	800 (700–900)	0.903
IUGR	24 (38.1%)	63 (36.0%)	62 (39.0%)	0.773
Multiple gestation	21 (32.3%)	60 (33.7%)	69 (40.1%)	0.180
Male gender	26 (40%)	92 (51.7%)	80 (46.5%)	0.684
Chorioamnionitis	5 (7.7%)	31 (17.4%)	45 (26.2%)	0.004
Antibiotics (mother)	40 (62.5%)	104 (62.3%)	101 (62.7%)	0.956
Prenatal steroids	52 (81.3%)	165 (96.5%)	157 (91.8%)	0.097
Cesarean section	44 (67.7%)	121 (68.8%)	101 (59.1%)	0.098
Apgar at 1 min ≤3	19 (30.2%)	26 (14.6%)	21 (12.3%)	0.004
Apgar at 5 min ≤6	11 (16.9%)	25 (14.3%)	25 (15.2%)	0.836
Umbilical artery pH	7.23 (7.13-7.30)	7.27 (7.21-7.32)	7.27 (7.17-7.32)	0.019
- Not 9performed	9 (13.8%)	39 (21.9%)	25 (14.5%)	0.133
- Mask/IPPV + PEEP	6 (9.2%)	65 (36.5%)	70 (40.7%)	<0.001
- ETI	44 (67.7%)	67 (37.6%)	70 (40.7%)	<0.001
- ETI + ChC + E	6 (9.2%)	7 (3.9%)	7 (4.1%)	0.195
- ET intubation	56 (86.2%)	121 (68.0%)	123 (71.5%)	0.143
- Duration (days)	4 (1–9)	2 (0–5)	2 (0–7)	0.005

IUGR: intrauterine growth restriction; ETI: endotracheal intubation; ChC: chest compression; E: epinephrine; IPPV: intermittent positive pressure ventilation; PEEP: positive end-expiratory pressure.

Median (IQR: 25^th^-75^th^ percentile), number (%).

Kruskal-Wallis or chi-square test.

**Table 2:** Morbidity and mortality in ELBW infants

	**Group 1**	**Group 2**	**Group 3**	p-value*
	**(1997–2000)**	**(2001–2005)**	**(2006–2009)**	
	**(N = 65)**	**(N = 178)**	**(N = 172)**	
CRIB	3 (1–8)	4 (2–7)	4 (2–8)	0.722
RDS	36 (55.4%)	99 (55.6%)	103 (59.9%)	0.432
Sepsis	28 (43.1%)	85 (47.8%)	65 (37.8%)	0.215
NEC	6 (9.2%)	12 (6.7%)	12 (7.0%)	0.644
IVH	18 (27.7%)	36 (20.2%)	43 (25.0%)	0.999
- Grades III/IV	11 (16.9%)	16 (9.0%)	18 (10.5%)	0.307
PVLM	1 (4.5%)	5 (5.6%)	4 (2.3%)	0.245
PDA	17 (26.2%)	56 (31.5%)	75 (43.6%)	0.004
- Surgical closure	1 (1.5%)	12 (6.7%)	18 (10.5%)	0.058
Parenteral nutrition (day)	13 (7–20)	9 (6–13)	8 (6–12)	0.009
Formula feed	24 (45.3%)	40 (25.2%)	17 (11.1%)	<0.001
Mortality**	23 (35.4%)	43 (24.2%)	40 (23.3%)	0.102
SWsBPD	38 (58.5%)	132 (74.2%)	129 (75.0%)	0.032
In survivors:	n = 42	n = 135	n = 132	
Mild BPD	8 (19.0%)	34 (25.2%)	39 (29.5%)	0.378
Moderate or severe BPD	4 (9.5%)	3 (2.2%)	3 (2.3%)	0.046
Weight at discharge (g)	2148 (2100–2250)	2060 (1900–2160)	2013 (1900–2193)	<0.001
Length of stay (days)	83 (64–102)	70 (59–83)	71 (59–85)	0.007
Weight gain (g/kg/day)	16 (14–18)	17 (15–19)	17 (15–19)	0.233

CRIB: clinical risk index for babies; RDS: respiratory distress syndrome; NEC: necrotizing enterocolitis; IVH: peri-intraventricular hemorrhage; PVLM: periventricular leukomalacia; PDA: patent ductus arteriosus; SWsBPD: survival without significant bronchopulmonary dysplasia.

Median (IQR: 25^th^-75^th^ percentile), number (%).

* Kruskal-Wallis or chi-square test; ** All deaths occurred in the NICU.

**Table 3:** Differences between survivors without significant BPD and deceased or surviving infants with significant BPD

	**SWsBPD**	**Deceased/Significant BPD**	p-value*
	**(n = 299)**	**(n = 116)**	
Year of birth			0.032
- 1997–2000	38 (58.5%)	27 (41.5%)	
- 2001–2005	132 (74.2%)	46 (25.8%)	
- 2006–2009	129 (75.0%)	43 (25.0%)	
Gestational age (w)	27.6 (26.1-29.4)	25.4 (24.4-27.0)	<0.001
Birth weight (g)	850 (750–935)	680 (589–780)	<0.001
IUGR	112 (38.5%)	37 (34.9%)	0.515
Multiple gestation	102 (34.1%)	48 (41.4%)	0.167
Male gender	135 (45.2%)	63 (54.3%)	0.094
Chorioamnionitis	48 (16.1%)	33 (28.4%)	0.004
Antibiotics (mother)	167 (59.6%)	78 (69.6%)	0.065
Prenatal steroids	277 (95.2%)	97 (84.3%)	<0.001
Cesarean section	213 (72.0%)	53 (45.7%)	<0.001
Apgar at 1 minute ≤3	29 (9.7%)	37 (32.5%)	<0.001
Apgar at 5 minutes ≤6	29 (9.7%)	32 (28.3%)	<0.001
Umbilical artery pH	7.26 (7.17-7.31)	7.29 (7.19-7.32)	0.165
Resuscitation			<0.001
- Not performed	67 (22.4%)	6 (5.2%)	
- Mask/IPPV + PEEP	121 (40.5%)	20 (17.2%)	
- ET intubation	102 (34.1%)	79 (68.1%)	
- ET Intubation +			
chest compression +			
epinephrine	9 (3.0%)	11 (9.5%)	
CRIB	4 (1–6)	9 (7–13)	<0.001
RDS	146 (48.8%)	92 (79.3%)	<0.001
Mechanical ventilation	184 (61.5%)	116 (100%)	<0.001
Sepsis	124 (41.5%)	54 (46.6%)	0.349
NEC	17 (5.7%)	13 (11.2%)	0.052
IVH	52 (17.4%)	45 (38.8%)	<0.001
- Grades III/IV	11 (3.7%)	34 (29.3%)	<0.001
PVLM	6 (3.1%)	4 (4.5%)	0.536
PDA	105 (35.1%)	43 (37.1%)	0.710
- Surgical closure	25 (8.4%)	6 (5.2%)	0.268
Severe arterial			
hypotension	10 (3.4%)	30 (26.1%)	<0.001

CRIB: clinical risk index for babies; IUGR: intrauterine growth restriction; ET: endotracheal; RDS: respiratory distress syndrome; NEC: necrotizing enterocolitis; IVH: peri-intraventricular hemorrhage; PEEP: positive end expiratory pressure; PVLM: periventricular leukomalacia; PDA: patent ductus arteriosus; SWsBPD: survival without significant BPD.

Median (IQR: 25^th^-75^th^ percentile), number (%).

* Mann–Whitney U or chi-square test.

**Table 4:** Odds ratios for significant associations between survival without significant BPD and related variables in ELBW infants

	**OR (95% CI)**	**p-value**
- Cesarean section	2.44 (1.38-4.33)	0.002
- Multiple gestation	0.46 (0.25-083)	0.011
- Level of resuscitation	0.36 (0.25-0.50)	<0.001
- Birth weight (1000 grams)	1.72 (1.51-1.96)	<0.001
- Mechanical ventilation (days)	0.97 (0.94-0.99)	0.015
- Intraventricular hemorrhage, grades III-IV	0.10 (0.04-0.23)	<0.001
- Severe arterial hypotension	0.17 (0.07-0.41)	0.001

Sample size: Included 409/415 newborns (99%); R^2^ = 0.646.

## Discussion

During the period studied, the data showed a trend toward a decrease in the death rate of ELBW infants, although this trend was not significant. Mild forms of BPD affected approximately 30% of the preterm infants during the study, whereas the incidence of severe forms of BPD declined, and the BPD-free survival rate increased from 58% (1997–2000) to 75% (2001–2009).

Chorioamnionitis and patent ductus arteriosus have been diagnosed more frequently in recent years. This increase in diagnosis may be due to an improvement in the accuracy of amniotic liquid analysis or heart ultrasound techniques, respectively. Extubation was performed earlier in recent years than it had been in the past, and PDA was more frequent. However, this was not followed by an increase in the diagnosis of BPD throughout the study period; in fact, the opposite trend was observed.

A change in the delivery room respiratory resuscitation strategy was introduced in 2001. With the increased use of CPAP since 2001, the need for endotracheal intubation and ventilation in the delivery room has diminished. The duration of mechanical ventilation has also decreased. The use of mechanical ventilation in premature infants may result in barotrauma, volutrauma and BPD [11]. Early surfactant therapy and the initiation of nasal CPAP in these infants significantly reduce the need for mechanical ventilation and the incidence of BPD [12]. In the COIN study [13], which included data from centers with a relatively recent history of using CPAP, a trend toward a lower rate of BPD was found in the CPAP group compared with the MV group. Many observational studies that compared primary CPAP and MV support found a decreased risk of developing BPD when CPAP was used as the primary means of ventilator support [14,15]. Together, these studies support a strong correlation between the use of MV and the development of BPD. The use of nasal CPAP appears to be a successful strategy for avoiding the need for mechanical ventilation in some infants, with the presumptive benefit of decreasing the risk of BPD [16]. The Columbian approach of using nasal CPAP in the delivery room in premature infants has resulted in a significant reduction in BPD. The recommended approach for ELBW infants with respiratory distress is to start CPAP in the delivery room immediately after stabilization, with intubation performed only for surfactant administration. In addition, adjustment of the supplemental oxygen intake is recommended to maintain pulse oximetry saturations between 85% and 93% in infants with a gestational age of less than 32 weeks [17-20]. In the SUPPORT trial, which involved ELBW infants, there was no significant difference between a strategy involving early CPAP and limited ventilation compared to one involving early intubation and surfactant administration within one hour after birth with respect to the rate of the composite primary outcome of death or BPD [21,22]. In secondary analyses, the CPAP strategy resulted in a lower rate of intubation than early surfactant treatment, both in the delivery room and in the NICU, which is similar to our findings. These data support the consideration of CPAP as an alternative to routine intubation and surfactant administration in preterm infants [23].

Other strategies that may be effective in reducing lung injury and subsequent BPD include the prevention of infection, early implementation of aggressive nutrition and treatment of a patent ductus arteriosus [24]. We began a policy of aggressive nutrition in 2001, with a significant decrease in the duration of parenteral nutrition and an increase in the amount of mother’s milk administered. These facts could have influenced the reduction of BPD incidence and length of stay with low weight at discharge, although the higher weight gain observed throughout the hospital stay did not represent a significant difference with low weight gain. However, Wilson [25] reported that sick VLBW infants allocated to an aggressive nutritional regimen exhibited better growth but similar survival and a similar incidence of BPD compared with a control group.

In 2006, we initiated a new mode of ventilation, namely, volume-guarantee (VG) ventilation, which reduces large tidal volumes, decreases the incidence of inadvertent hyperventilation, reduces the duration of mechanical ventilation and pneumothorax [26], and reduces proinflammatory cytokine levels [11]. Furthermore, VG decreases the expression of early inflammatory markers to a greater extent than high-frequency oscillatory ventilation [27]. Therefore, VG may decrease the incidence of BPD; however, in our study, there was no reduction in the BPD rate after 2006, although the duration of mechanical ventilation remained low.

Cesarean section was associated with elective preterm delivery, and vaginal delivery was associated with emergency deliveries and chorioamnionitis. For this reason, preterms born by cesarean section have better prognoses and typically do not require aggressive prenatal support [28].

This study has some limitations. The data were collected from a database but were introduced prospectively during the study period. The most important changes during this period were the introduction of respiratory assistance in the delivery room, the initiation of a protocol of aggressive nutrition and the practice of VG ventilation. However, other changes could have influenced the neonatal prognosis. The strength of the study was the use of a large number of ELBW infants (415) who were born at the same hospital, thus yielding similar baseline characteristics.

## Conclusions

Over the past 13 years, the morbidity of ELBW infants has not changed; however, the rate of SWsBPD has increased (75.0%). Delivery by caesarean section and reductions in the endotracheal intubation rate and the duration of mechanical ventilation are protective factors that could reduce the frequency of SWsBPD in these infants.

## Abbreviations

BPD: bronchopulmonary dysplasia; CPAP: continuous positive airway pressure; ELBW: extremely-low-birth-weight; IPPV: intermittent positive pressure ventilation; IQRL: interquartile range; nCPAP: nasal continuous positive airway pressure; NICU: neonatal intensive care unit; PEEP: positive end-expiratory pressure; PIP: peak inspiratory pressure; PN: parenteral nutrition; RDS: respiratory distress syndrome; SWsBPD: survival without significant bronchopulmonary dysplasia.

## Competing interests

The authors declare that they have no competing interests.

## Authors’ contributions

FB and JFA shared the primary responsibility for protocol development, patient screening, patient enrollment, outcome assessment, preliminary data analysis and manuscript writing. XME, JMRM and MDSR were responsible for patient screening and participated in the protocol development. XCE contributed to the analytical framework of the study and to the writing of the manuscript. All authors read and approved the final manuscript.

## Pre-publication history

The pre-publication history for this paper can be accessed here:

http://www.biomedcentral.com/1471-2431/12/63/prepub
